# An opportunity for fertility preservation in neurosurgical spinal ependymoma patients: a single center review

**DOI:** 10.3389/frph.2025.1730257

**Published:** 2026-01-12

**Authors:** Madison J. Michles, Christian B. Schroeder, Lauren R. Sugarmann, Felicia W. Sun, Morgan Kruzan, Luce Kassi, May-Tal Sauerbrun-Cutler, Ziya L. Gokaslan, Patricia Zadnik Sullivan

**Affiliations:** 1 Department of Neurosurgery, Warren Alpert Medical School of Brown University, Rhode Island Hospital, Providence, RI, United States; 2 Division of Reproductive Endocrinology and Infertility, Women and Infants Hospital of Rhode Island, Providence, RI, United States

**Keywords:** fertility preservation, neuro-oncology, neurosurgery, reproductive endocrinology and infertility, spinal ependymoma

## Abstract

Spinal ependymomas are intradural intramedullary tumors that present frequently in adults with a bimodal peak at 25–29 years and again at 45–59 years. The risk of progressive neurological deficit in these patients creates the need for surgical resection and radiation therapy. Myxopapillary ependymomas specifically occur at the conus medullaris within the lumbar spine, and these treatments can have a significant impact on patient fertility. However, recent reports illustrate that a vast majority of adults with cancer receive inadequate fertility preservation education, counseling, or resources, and providers tend to cite barriers such as lack of knowledge about fertility preservation options or referrals as well as discomfort with broaching the topic. This review aims to explore the extent to which fertility preservation counseling was offered by a neurosurgery department at a single institution to patients with ependymomas and the subsequent utilization of fertility preservation services. A retrospective review of our local spine tumor database from 2015 to 2025 identified 15 patients who underwent surgery for spinal ependymomas at Rhode Island Hospital. Patients who were outside of child-bearing years (<18 years to >44 years) were excluded (*N* = 4). Medical records were searched for references to fertility counseling within the notes as well as subsequent receipt of patient services. This cohort consisted of 3 female and 8 male patients with a median age of 42 years. Two patients underwent postoperative radiation treatment. Only one patient in the cohort received any type of fertility preservation counseling or discussion of fertility-related risks of treatment and was referred for sperm banking services. These results shed light on a gap in care regarding fertility in neurosurgical oncology. Previous research suggests that there are significant benefits of fertility preservation counseling and REI referral in every specialty that provides oncology care. This implies that, as a part of the oncological care team, neurosurgeons may be missing a critical opportunity to help their patients achieve goals that extend past their cancer journeys, and further development of protocols for fertility preservation counseling are needed in order for neurosurgeons to provide quality care beyond the doors of the operating room.

## 1 Introduction

Ependymomas are tumors of the central nervous system whose location is largely dictated by patient age. In adults, ependymomas are intradural intramedullary tumors of the spine, and their incidence follows a bimodal distribution of 25–29 and 45–59 years of age at presentation (1). These tumors carry a significant risk of neurological deficit via spinal cord compression, and therefore surgical resection is a mainstay of treatment. Complete excision can be curative, but recurrence is possible, especially when complete resection is not feasible (2). In these cases, patients usually receive radiotherapy, although the efficacy and optimal dose remain controversial (3). Chemotherapy, usually temozolomide, is occasionally used in the event of recurrent ependymomas refractory to local treatment (4).

Some histological subtypes of spinal ependymomas tend to occur at specific locations. Myxopapillary ependymomas, for example, have a propensity for the conus medullaris and filum terminale (1). As such, complete resection of this type of ependymoma can be challenging and adjuvant radiotherapy may be required (5).

When ependymomas occur in the lumbosacral spine, treatment options in this area can be associated with threats to fertility in both sexes. In patients with a uterus and ovaries, both radiotherapy directly to the pelvic area and scattered radiation can result in permanent loss of oocytes and impairment of ovarian function (6). In patients with testicles, even low-dose radiation is associated with a decrease in functional spermatogonia, although this is more likely to be temporary than the aforementioned reduction in ovarian function (7). Surgery alone is associated with fertility-related risks for male patients: anterior lumbar surgery carries a well-known risk of retrograde ejaculation (8). For both sexes, cytotoxic chemotherapeutics are associated with gonadotoxicity (6). Despite these risks, preoperative discussions rarely include a review of the patient's sexual function and goals for family planning.

Despite the well-defined risks of these treatments on patient fertility, recent reports reveal that a vast majority of adults with cancer receive inadequate fertility preservation education, counseling, or resources (9–12). The American Society of Clinical Oncology's guidelines regarding fertility preservation emphasize the importance of fertility counseling from all oncologic providers, and patients report benefits from discussing fertility multiple times during their cancer journeys (13). Cancer survivors often cite fertility complications as one of the most distressing outcomes of cancer treatment, and many patients report feeling that their providers perceived fertility issues as a low priority compared to cancer treatment (14). Maintaining fertility in ependymoma patients in particular should be discussed because spinal ependymomas tend to have a favorable long-term prognosis (1), meaning that it is not only appropriate but essential for providers to consider their patients' lives after conclusion of treatment.

The goal of this study is to examine the extent to which fertility counseling occurred and subsequent fertility preservation services were utilized by a cohort of spinal ependymoma patients in the authors' local spine tumor database. A narrative literature review was also performed in order to shed light on the potential barriers to effective fertility counseling by the neurosurgical team.

## 2 Methods

A retrospective review of the authors’ local spine tumor database from 2015 to 2025 identified 15 patients who underwent surgery for spinal ependymomas at Rhode Island Hospital. Patients who were outside of child-bearing years (age < 18 years or >44 years) were excluded (*N* = 4). Medical records were searched for references to fertility counseling within the notes as well as subsequent receipt of patient services. A narrative literature review was also performed via PubMed utilizing search terms such as “oncofertility,” “spine surgery fertility preservation,” and “cancer fertility counseling.”

## 3 Results

This cohort consisted of 3 female and 8 male patients with a median age of 42 years. Full demographic data is outlined in Table 1. Two patients underwent radiation treatment (one intensity-modulated radiation therapy and one proton-based radiation therapy) and no patients received chemotherapy. Two patients required reoperation: one for a surgical site infection requiring debridement and one for a wound breakdown requiring a washout (Table 2). Only one patient in the cohort received any type of fertility preservation counseling or discussion of fertility-related risks of treatment and was referred for sperm banking services. This patient was advised of a temporary decrease in fertility for up to a year by the neurosurgeon, and the patient did not ultimately utilize the referral to urology and fertility services.

**Table 1:** Demographic data.

Case No.	Age at Date of Surgery (yrs), Sex	Tumor Levels	Radiotherapy	Fertility Counseling
1	25, F	L1-L3	N/A	No
2	40, F	L4	N/A	No
3	57, M	L1-L2	N/A	No
4	30, M	T12-L1, L5-S1	IMRT	No
5	35, F	C2-C5	N/A	No
6	43, M	T10-T11	N/A	No
7	12, M	T7-T10	N/A	No
8	29, M	C7-T1	N/A	No
9	40, M	C5-C7	N/A	No
10	55, M	C4	N/A	No
11	31, M	L3-L4	Proton-based	Yes

**Table 2:** Surgical details, complications, and reoperations.

Case No.	Surgical approach	Complications	Reoperations
1	Posterior	Unplanned neurological deficit	N/A
2	Posterior	Wound breakdown, unplanned neurological deficit	Wound revision/washout
3	Posterior	N/A	N/A
4	Posterior	N/A	N/A
5	Posterior	N/A	N/A
6	Posterior	Unplanned neurological deficit	N/A
7	Posterior	N/A	N/A
8	Posterior	Surgical site infection, unplanned neurologic deficit	Surgical site debridement
9	Posterior	Construct instability without breakage	N/A
10	Posterior	Construct instability without breakage	N/A
11	Posterior	N/A	N/A

## 4 Discussion

As cancer treatments become increasingly efficacious and cancer survivability improves, oncological care should increase in scope accordingly to prepare patients for life after cancer. Prioritizing patients' fertility goals after oncological treatment is a major component of what previous research has termed the new paradigm of cancer care: quality survival rather than survival alone (10). In our cohort, only one male patient out of eight was advised of any potential fertility impacts of surgery or radiotherapy treatments and was subsequently referred to fertility preservation services. None of the female patients (*n* = 3) were offered referral to a fertility specialist. We believe, in accordance with fertility experts and previous research, that we as a neurosurgery department are missing a critical opportunity for patient care.

### 4.1 Surgical impacts on fertility

The gold standard of treatment for spinal ependymomas is gross total resection (GTR) to both improve symptoms and mitigate the potential for CSF seeding (4). Complex local anatomy at the conus medullaris and cauda equina can complicate resection and impact neurological outcomes (15). In one study, 23% of spinal ependymoma patients reported some degree of sexual dysfunction postoperatively (16). In another study, 15% of male spinal ependymoma patients reported erectile dysfunction or decreased sensation postoperatively, 5% of which were refractory to treatment (17). While surgical management of spinal ependymomas has fewer fertility-related risks than systemic treatments, such as chemotherapy and radiotherapy, patients with testes should still be counseled on the potential for these sexual side effects.

### 4.2 Radiotherapy impacts on fertility

Complete surgical resection of ependymomas can be made challenging in locations such as the conus medullaris and cauda equina. As a result, patients with tumors in these areas may receive adjuvant regional radiotherapy (5). Additionally, a 2024 study found that adjuvant radiotherapy was more common in ependymoma patients with the myxopapillary subtype, a finding which may have been related to cerebrospinal fluid dissemination secondary to disruption of the thin tumor capsule (18). Patients receiving radiotherapy, regardless of sex or gender, are at risk of gonadal radiation exposure, which reduces the number and function of ovarian follicles and spermatogonia (6). The ovaries are known to be very sensitive to damage from radiotherapy, and the dose required to destroy 50% of immature ovarian follicles has been calculated to be <2 Gy, which is well below the typically prescribed dose ranges for spinal ependymomas (19–21). Additionally, ovarian exposure to radiation has been associated with premature menopause, and the extent of ovarian damage seems to be inversely correlated with patient age (19, 22). Additionally, previous research suggests that a history of abdominal or pelvic radiation was associated with increased rates of uterine dysfunction and subsequent preterm labor, low birth weight, and placental abnormalities (19). Similarly, the testes are extremely radiosensitive, with spermatogenesis impairment occurring at radiation doses of >0.1 Gy, and are frequently damaged due to scattered radiation during treatment of nearby tissue, even with testicular shielding (23, 24).

In the event of recurrence or metastatic seeding, a complication which can result from resection of these intradural tumors, patients can receive high dose craniospinal radiotherapy of 30–36 Gy (4, 21). Craniospinal radiation has been associated with hypothalamic-pituitary-gonadal (HPG) dysregulation and gonadotropin deficiency, which can be associated with varying degrees of infertility for patients with ovaries and testes alike (24–27). Further, craniospinal radiation has been identified as a risk factor for later miscarriage when the ovaries are not shielded (19).

### 4.3 Chemotherapeutic impacts on fertility

The use of chemotherapy in spinal ependymomas may be indicated for patients with recurrent disease who are not candidates for surgical excision or radiotherapy, although its efficacy remains unclear (4, 28). Agents known to penetrate the blood-brain barrier, such as temozolomide, are the traditional choice (4). Temozolomide and other alkylating agents, such as platinum derivatives, are associated with impairment of ovarian and testicular function and therefore considered to be moderately to highly gonadotoxic (6). While the impacts of temozolomide specifically on fertility are less well-characterized and limited to small, single institution studies, alkylating agents in general have been shown to have a dose-dependent cytotoxic effect on gonadal tissue in the treatment of cancers such as lymphomas and leukemias (29–32).

In patients with ovaries, alkylating chemotherapy has been linked to direct oocyte destruction and follicle depletion as well as ovarian fibrosis and blood vessel damage (30). This damage can result in premature ovarian failure in up to 25% of patients (33). In patients with testes, alkylating chemotherapy can result in reduced spermatogonia production or viability, with one study estimating the prevalence of prolonged azoospermia in 90%–100% of patients receiving treatment (33). While patients with testes are more likely to achieve recovery of gonadal function than patients with ovaries due to the fixed number of ovarian follicles, it can take up to three years or more for spermatogenesis to recover after chemotherapy (34). However, patients have been reported to recover spermatogenesis even after receiving a previously-considered irreversibly toxic dose of cisplatin (35), so predicting to what extent chemotherapy will impact fertility in any one patient remains challenging.

As previously discussed, achievement of gross total resection is often difficult with spinal myxopapillary ependymomas, and therefore these patients may be at a higher risk of recurrent and/or residual disease: a 2023 study found that almost 78% of patients with subtotal resections underwent reoperation for recurrence at a median of 32 months after initial resection (36). Given that the incidence of these tumors is increased in 25–29 year-old patients, this implies that the majority of these patients would still be within reproductive age should recurrence occur (1). Although our patient population had an older median age of 42 years, male patients would still be considered within reproductive age and could experience a decrease in spermatogonia count, quality, or motility as a result of chemotherapy treatment (37).

### 4.4 Glucocorticoid impacts on fertility

High doses of glucocorticoids, such as dexamethasone, can also potentially affect fertility. Physiologically, glucocorticoids can inhibit the release of gonadotropin-releasing hormone (GnRH) at the level of the hypothalamus (38, 39). This inhibition can subsequently lead to a decrease in the production of luteinizing hormone (LH) from the pituitary gland (40). Glucocorticoids exert their cellular effects by binding to the glucocorticoid receptor (GR), which can be found in both testis and ovaries. The presence of GR on gonadal cells suggests a direct influence on reproductive function (41, 42). Glucocorticoids can act directly at the gonadal level by inhibiting steroid hormone production or inducing glucocorticoid-mediated apoptosis (43, 44). This disruption in the hypothalamus-pituitary-ovary (HPO) axis may decrease the production of FSH and LH, estradiol production, follicular growth and ultimately affect fertility (45). Despite the theoretical suppression of hormones in the setting of high dose steroid use, it has not been demonstrated to significantly impact oocyte yield in IVF cycles (46). Therefore, fertility preservation procedures should not be delayed for patients who are on steroids.

### 4.5 Considerations for fertility preservation utilization

There remains no clear consensus on the average length of treatment for spinal ependymomas (1). For patients with primary spinal ependymoma, preoperative neurological status guides the urgency of treatment initiation: patients with more severe neurologic deficits are treated more emergently. Although smaller tumor size and better preoperative neurological function are associated with improved postoperative outcomes, spinal ependymomas are generally considered to follow an indolent course, meaning that surgical treatment could be temporarily delayed in patients with stable neurological status to allow for fertility preservation treatment (1, 3). Additionally, prior to surgery, patients often receive high dose dexamethasone to mitigate spinal cord compression and subsequent neurological symptoms.

While it is well-known that increased age is associated with decreases in fertility for patients with ovaries, patients with stable neurological status should be able to undergo one to two cycles of oocyte collection (four to six weeks in total) immediately prior to surgery without compromising their neurosurgical care. Postoperatively, patients can begin the implantation process once they are able to comfortably lie supine. Therefore, patients should not be impacted by increasing age except in the case of longer-term radiotherapy and chemotherapy for recurrent disease.

### 4.6 Recommendations for fertility counseling in neurosurgery

Previous research and reproductive endocrinologists stress the importance of having conversations regarding fertility preservation at multiple points during patients' cancer journeys (10, 13, 14, 47). Cancer patients requiring neurosurgery enter the healthcare system through different pathways, some of which may not involve consultations with oncological specialists. As a result, these patients may not have had prior opportunities to discuss potential side effects on their fertility. Reproductive endocrinologists working with cancer patients emphasize the importance of being transparent about the fertility risks of treatment. Fertility preservation treatments for oncology patients are covered by commercial insurance in 15 states but are only covered by non-commercial insurance in 2 states (48). No insurance coverage is a common barrier to care. For women, the most commonly used options for fertility preservation are oocyte or embryo cryopreservation, which can take on average two to three weeks. For men, the process of spermatogonia collection and cryopreservation can be completed in a day (49). Though fertility preservation should not delay urgent oncological treatment, the ideal time for these options is before any surgical or chemoradiation therapy in order to mitigate the potential for radiation damage to reproductive tissue. However, pre-treatment referrals are not always possible, and in these cases, a balance must be struck between prompt oncological treatment and fertility preservation goals, re-emphasizing the need for strong multidisciplinary collaboration. In cases where emergent spinal surgery is required, and after postoperative surgical clearance, patients should still be offered referral to a fertility specialist prior to any additional chemoradiation therapies. Reproductive endocrinologists and male andrologists emphasize the importance of referring all patients who may be interested in fertility preservation. This also includes patients who may feel ambivalent about their reproductive goals and who might still benefit from counseling on their available options. In all cases, shared decision-making between patient and provider is essential in affirming and realizing patients' fertility goals.

Prior research has indicated that quality of life outcomes in young cancer patients are influenced by the potential for future fertility (50, 51). The possibility of retained future fertility has been associated with improved coping with diagnosis as well as upward trends in physical and psychological quality of life (51). Interestingly, regardless of whether patients chose to pursue fertility preservation, patients tended to have greater satisfaction when their oncological providers discussed fertility preservation topics with them (52). Further, recent studies revealed that discussion and consideration of fertility preservation among cancer patients did not significantly influence patients' oncology treatment choices (52), and that oncological outcomes were similar regardless of whether patients elected to pursue fertility preservation (53). Taken together, this implies that satisfaction and quality of life outcomes in cancer patients can be improved by discussing and prioritizing the possibility of fertility preservation regardless of patients' ultimate decisions, and thoughtful and timely interventions to preserve future fertility should not compromise quality oncological care.

**Figure 1:**
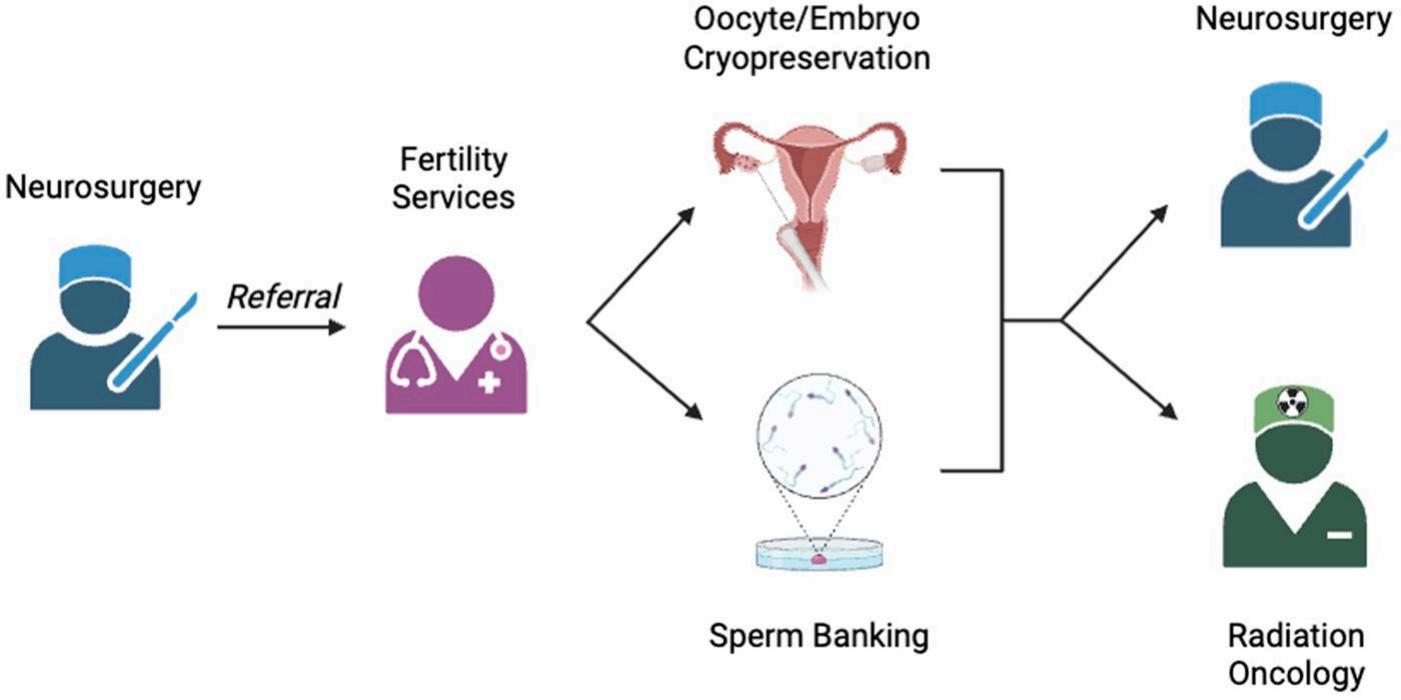
Proposed workflow for maximizing fertility preservation in neurosurgical oncology patients.

Previous studies have specifically identified surgeons as being unlikely to counsel patients regarding fertility and refer patients to fertility specialists due to the view that such conversations were beyond the scope of the surgeon's practice (10, 47). However, without a clear system to ensure that oncologists consistently discuss how recommended treatments may impact fertility, patients may miss out on crucial information needed for their care. Additionally, referral to a fertility specialist will also provide more in-depth counseling on the available options, even for patients who initially had the conversation with their neurosurgeons. Patients who receive fertility-related counseling from both the oncological team and the fertility preservation team report less decisional regret, and just being referred for a fertility preservation consultation has been associated with reduced decisional conflict (14). Further, in the initial aftermath of a cancer diagnosis, patients may have been emotionally unable to participate in or initiate such a conversation and could benefit from another discussion and the opportunity for a referral to fertility preservation experts. Therefore, neurosurgeons should be a part of this discussion to ensure that each patient is offered the opportunity to make informed decisions about their cancer treatments as well as their lives afterwards (Figure 1).

## 5 Conclusions

This retrospective cohort study reveals that patients with spinal ependymomas tend not to be counseled regarding potential fertility impacts of treatment and subsequently tend not to receive referrals to fertility preservation services. Therefore, there exists a serious need for every member of the oncology team, including neurosurgeons, to participate in the conversation regarding fertility risks and preservation options with patients. A collaborative and interdisciplinary effort with reproductive specialists could improve patient outcomes and satisfaction during cancer treatment and beyond.

## Data Availability

The raw data supporting the conclusions of this article will be made available by the authors, without undue reservation.
